# Fetal dose conversion factor for fetal computed tomography examinations: A mathematical phantom study

**DOI:** 10.1002/acm2.12154

**Published:** 2017-08-11

**Authors:** Yuta Matsunaga, Ai Kawaguchi, Masanao Kobayashi, Shoichi Suzuki, Yasuki Asada, Kiyoshi Ito, Koichi Chida

**Affiliations:** ^1^ Graduate school of Medicine Tohoku University Aoba‐ku, Sendai Japan; ^2^ Department of Imaging Nagoya Kyoritsu Hospital Nagoya Japan; ^3^ Department of Radiology TOYOTA Memorial Hospital Toyota Japan; ^4^ School of Health Sciences Fujita Health University Toyoake Japan; ^5^ Department of Disaster Obstetrics and Gynecology International Research Institute of Disaster Science (IRIDeS) Tohoku University Aoba‐ku, Sendai Japan

**Keywords:** computed tomography, dose‐length product, fetal dose, pregnant woman

## Abstract

This study aimed to examine the relationship between fetal dose and the dose–length product, and to evaluate the impact of the number of rotations on the fetal doses and maternal effective doses using a 320‐row multidetector computed tomography unit in a wide‐volume mode. The radiation doses for the pregnant woman and the fetus were estimated using ImPACT CT Patient Dosimetry Calculator software for scan lengths ranging from 176 to 352 mm, using a 320‐row unit in a wide‐volume mode and an 80‐row unit in a helical scanning mode. In the 320‐row unit, the fetal doses in all scan lengths ranged from 3.51 to 6.52 mGy; the maternal effective doses in all scan lengths ranged from 1.05 to 2.35 mSv. In the 80‐row unit, the fetal doses in all scan lengths ranged from 2.50 to 3.30 mGy; the maternal effective doses in all scan lengths ranged from 0.83 to 1.68 mSv. The estimated conversion factors from the dose–length product (mGy・cm) to fetal doses (mGy) for the 320‐row unit in wide‐volume mode and the 80‐row unit in helical scanning mode were 0.06 and 0.05 (cm^−1^) respectively. While using a 320‐row MDCT unit in a wide‐volume mode, operators must take into account the number of rotations, the beam width as automatically determined by the scanner, the placement of overlap between volumetric sections, and the ratio of overlapping volumetric sections.

## 1 INTRODUCTION

Skeletal dysplasias are a heterogeneous and complex group of conditions that affect the growth and development of the bone and cartilage, and result in various anomalies in the shape and size of the skeleton.^1^, ^2^ The prevalence of these dysplasias in neonates has been estimated at approximately 2.44 per 10,000.^3^ Conventionally, ultrasonography and magnetic resonance imaging have been used for the prenatal diagnosis of severe skeletal dysplasia.^1^, ^4^, ^5^ Computed tomography (CT) has been found to be highly effective in diagnosing fetal skeletal dysplasia^6^, ^7^; however, the risk of radiation exposure to the fetus and mother cannot be avoided since CT employs X‐rays. Fetuses are more radiosensitive than adults and children.^8^, ^9^ Therefore, when pregnant women undergo CT examination, it is important to know the associated fetal radiation risk.

CT units generate patient–dose indexes of the volume CT dose index (CTDI_vol_) and the dose–length product (DLP) that are measured in 16‐ and 32‐cm diameter acrylic phantoms. The scientific literature contains only limited data regarding effective dose/DLP conversion factors, and there are, to our knowledge, no studies investigating the fetal dose/DLP conversion factors for fetal CT examination.^10^, ^11^, ^12^ Elucidating the relationship between fetal doses and DLP could assist in estimating the fetal dose in clinical practice.

Recently, a 320‐row multidetector CT (MDCT) unit has become available that allows axial volume‐scanning with a 160‐mm range. For imaging neonates and small children, volume‐scanning is of potential great advantage as the entire scan can be performed in one rotation. Because there is no over‐ranging associated with axial volumetric scanning, this may reduce the patient's radiation dose.^13^ In addition, a volume‐scanning mode in a 320‐row MDCT unit has been used in fetal CT.^14^ However, Matsunaga et al.^14^ reported that operators must take into account the number of rotations, beam width, and overlap between volumetric sections, because the volumetric scanning mode, using multiple contiguous sections in the 320‐row MDCT unit, caused wide overlapping of the volumetric sections. Despite this concern, the relationship between the radiation dose and the number of rotations in a volume‐scanning mode has not been described in previous reports.

This study examined the relationship between fetal dose and DLP, and evaluated the impact of the number of rotations on the fetal doses and maternal effective doses using a 320‐row MDCT unit in a wide‐volume mode.

## 2 METHODS

**Figure 1:**
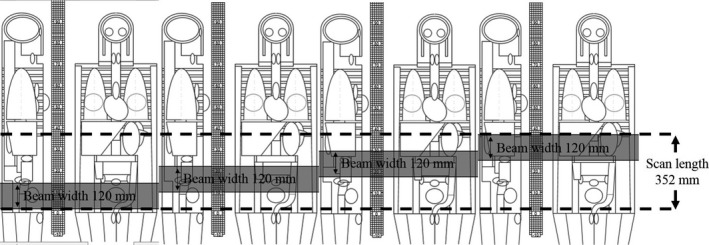
A graphic illustration depicting the calculation method using the 320‐row multidetector computed tomography unit in wide‐volume mode and a scan length of 352 mm (beam width 120 mm * number of rotations 4).

The radiation doses for a theoretical pregnant woman and fetus were estimated using the ImPACT CT Patient Dosimetry Calculator software (2011, Version 1.0.4; Scanner Evaluation Centre of the United Kingdom National Health Service). This CT dosimetry software makes use of the National Radiological Protection Board's Monte Carlo dose data sets produced in report SR250 (Health Protection Agency Centre for Radiation, Chemical and Environmental Hazards, Didcot, UK).^15^ This report provides normalized organ dose data for irradiation of a mathematical phantom. The radiation doses from a 320‐row MDCT unit (Aquilion ONE, Toshiba Medical System Corporation, Otawara, Japan) and an 80‐row MDCT unit (Aquilion PRIME, Toshiba Medical System Corporation, Otawara, Japan) were estimated. All scan parameters used in this study were based on those reported by Matsunaga et al.^14^ (Table 1). The number of rotations and beam width cannot be adjusted manually in the 320‐row MDCT unit in a wide‐volume mode and were instead automatically determined by the scan length. The scan length is generally based on the fetus length; thus, scan lengths differ due to differences in fetal size. The radiation doses for the pregnant woman and the fetus were estimated by using the following scan lengths (beam width * number of rotations): 176 mm (120 mm * 2), 184 mm (128 mm * 2), 204 mm (140 mm * 2), 232 mm (160 mm * 2), 264 mm (120 mm * 3), 276 mm (128 mm * 3), 306 mm (140 mm * 3), 348 mm (160 mm * 3), and 352 mm (120 mm * 4). The scan length was entered separately from the number of rotations in the spreadsheet (Fig. 1), taking into account the overlap between volumetric sections. The sum of radiation doses from the number of rotations was defined as the estimated radiation dose. This method of estimating radiation doses was based on that used in the study by Matsunaga et al.^16^ The 80‐row MDCT unit employed a helical scanning mode. Since the 80‐row MDCT unit uses a high‐pitch factor, there is no overlap between the helical sections.^14^ Therefore, the exposure parameters presented in Table 1, including scan length, were entered into a spreadsheet, and the radiation doses for the 80‐row MDCT unit were estimated. The scan lengths for the 80‐row MDCT unit were 176, 184, 204, 232, 264, 276, 306, and 348 mm. The setting of 352 mm was omitted, because 348 and 352 mm would both have been rounded to 350 mm by the CT dosimetry software. In this study, the radiation dose to the uterus was used as an estimate of that to the fetus.

**Table 1:** Scan parameters entered into the spreadsheet in the 320‐row MDCT unit and 80‐row MDCT unit

Beam width (mm)	Number of rotations	Scan length (mm)	Tube voltage (kV)	Tube current (mA)	Pitch factor	Gantry rotation time (s)	CTDI_vol_ (mGy)^b^	DLP (mGy cm)
Wide‐volume mode in 320‐row MDCT								
120	2	176 (175)^a^	100	103	1.00	0.5	2.6	62
128	2	184 (185)^a^					2.6	68
140	2	204 (205)^a^					2.7	74
160	2	232 (235)^a^					2.7	88
120	3	264 (270)^a^					2.6	93
128	3	276 (275)^a^					2.6	102
140	3	306 (310)^a^					2.7	111
160	3	348 (350)^a^					2.7	132
120	4	352 (350)^a^					2.6	124
Helical scanning mode in 80‐row MDCT								
40	1	176 (175)^a^	100	160	1.39	0.5	2.9	52
		184 (185)^a^						54
		204 (205)^a^						58
		232 (235)^a^						67
		264 (270)^a^						78
		276 (275)^a^						80
		306 (310)^a^						90
		348 (350)^a^						101

MDCT, multidetector computed tomography; CTDI, computed tomography dose index; DLP, dose–length product.

a are values entered into the spreadsheet. Because the mathematical phantom used in this study was divided from head to mid‐thigh into 208 axial slabs of 5 mm in thickness, the scan lengths in the spreadsheet must be entered in 5‐mm intervals.

b CTDI_vol_ is reported for the 32‐cm CTDI phantom.

In this study, the effective dose was derived using only the female organs; however, the recommendations of the International Commission on Radiological Protection (ICRP)^9^ define effective doses for the average male and female patient. The ICRP created an important qualifier that effective dose is not to be applied to the individual patient since, among other reasons, the risk factors used were an average over a reference adult population. It must be noted that the effective dose derived in this study is an approximation of the actual effective dose. Kawaura et al.^17^ evaluated the female effective dose using a similar method.

The authors modified the ImPACT software to evaluate the radiation doses of the Toshiba Aquilion ONE and Toshiba Aquilion PRIME CT units, since it is not included by default in this software. The modification of this software was based on that used in previous studies.^18^, ^19^

## 3 RESULTS

**Figure 2:**
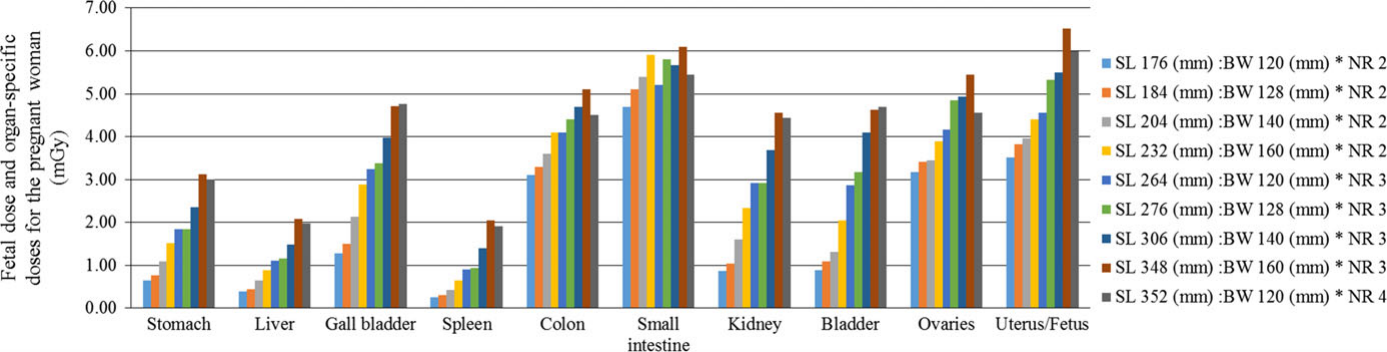
The fetal dose and organ‐specific doses for the pregnant woman in the 320‐row MDCT unit in a wide‐volume mode. BW, beam width; MDCT, multidetector computed tomography; NR, number of rotations; SL, scan length.

**Figure 3:**
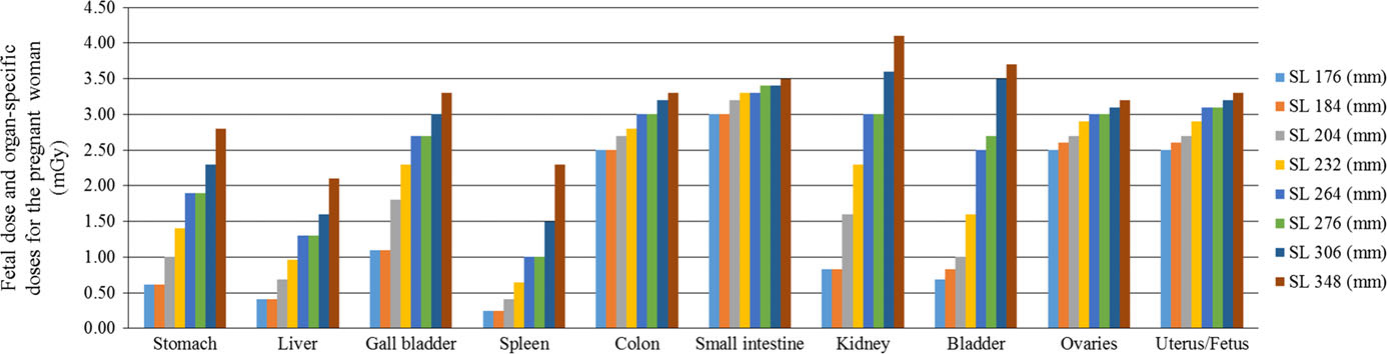
The fetal dose and organ‐specific doses for the pregnant woman in the 80‐row MDCT unit in a helical scanning mode. MDCT, multidetector computed tomography; SL, scan length.

The organ‐specific doses for the pregnant woman and the fetal dose in the 320‐row MDCT unit in a wide‐volume mode and the 80‐row MDCT unit in a helical scanning mode are listed in Figs. 2 and 3 respectively. In the 320‐row MDCT unit, as the scan length increased, the fetal doses increased. However, an increase in the number of rotations did not necessarily correspond to an increase in the fetal doses, e.g., the fetal dose did not increase between 348 mm (160 mm * 3) and 352 mm (120 mm * 4). The radiation dose to the organs in the pregnant woman included in the scan range (e.g., the colon, small intestine, and ovaries) also yielded similar results. The small intestine doses were the largest in each scan length, except at the uterus/fetus.

In the 80‐row MDCT unit, as the scan length increased, the fetal doses increased. A similar trend was observed in the radiation dose to the organs in the pregnant woman.

The ratios of the fetal doses (mGy) to DLP (mGy・cm) are shown in Table 2. The ranges of the ratios of the fetal doses (mGy) to DLP (mGy・cm) in the 320‐ and 80‐row MDCT unit were 0.05–0.06 and 0.03–0.05 respectively.

**Table 2:** The ratio of the fetal doses (mGy) to DLP (mGy・cm) in the 320‐row MDCT unit in a wide‐volume mode and 80‐row MDCT unit in a helical scanning mode

Scan length (mm)	Wide‐volume mode 320‐row MDCT				Helical scanning mode 80‐row MDCT		
	Beam width (mm) * Number of rotations	DLP (mGy cm)	Fetal dose (mGy)	Fetal dose/DLP	DLP (mGy cm)	Fetal dose (mGy)	Fetal dose/DLP
176	120*2	62	3.51	0.06	52	2.50	0.05
184	128*2	68	3.82	0.06	54	2.60	0.05
204	140*2	74	3.96	0.05	58	2.70	0.05
232	160*2	88	4.40	0.05	67	2.90	0.04
264	120*3	93	4.55	0.05	78	3.10	0.04
276	128*3	102	5.32	0.05	80	3.10	0.04
306	140*3	111	5.50	0.05	90	3.20	0.04
348	160*3	132	6.52	0.05	101	3.30	0.03
352	120*4	124	5.99	0.05			

DLP, dose–length product; MDCT, multidetector computed tomography.

In the 320‐row MDCT unit, the maternal effective dose for each scan length (listed in Fig. 2) was 1.05, 1.15, 1.29, 1.54, 1.65, 1.81, 2.01, 2.35, and 2.17 mSv respectively. In the 80‐row MDCT unit, the maternal effective dose for each scan length (listed in Fig. 3) was 0.83, 0.85, 0.99, 1.14, 1.33, 1.34, 1.51, and 1.68 mSv respectively.

## 4 DISCUSSION

Exposure of the fetus was considered as whole‐body exposure, because the scan length in fetal CT is generally based on the fetus’ length. In contrast, the DLP can be used to evaluate the radiation risk while taking into account the scan length and the overlapping of volume‐scanning. There is a possibility that the approximate fetal dose may be estimated by multiplying the displayed DLP with a constant conversion factor. Based on the ratio of the fetal doses (mGy) to DLP (mGy・cm) in Table 2, the estimated conversion factors from DLP (mGy・cm) to fetal doses (mGy) for the 320‐row MDCT unit in wide‐volume mode and the 80‐row MDCT unit in helical scanning mode were 0.06 and 0.05 (cm^−1^), respectively; to be conservative, it is recommended that the highest conversion factors be used. In the 320‐row MDCT unit, the fetal doses estimated by multiplying the displayed DLP with the conversion factor calculated in this study (5.91 mGy) were almost the same as those derived from physical measurements using thermoluminescent dosimeters in a previous study (5.50 mGy).^14^ Furthermore, the conversion factor calculated in this study yielded similar results for the 80‐row MDCT unit. It may be important to understand that these factors will be influenced by the X‐ray tube voltage, scan range, and patient size. These findings suggest that if a dosimeter or dose simulation software is not available in clinical practice, it is possible to estimate the approximate fetal dose in fetal CT by calculating the DLP at the time and multiplying it by the conversion factors (0.06 and 0.05) determined in this study.

As the scan length and number of rotations increased from 176 to 348 mm and 2 to 3 scans, respectively, the fetal doses and maternal effective doses also increased. However, when the scan length and number of rotations increased from 348 to 352 mm and 3 to 4 scans, respectively, the fetal doses and maternal effective doses decreased. This occurred because the ratio of overlapping volumetric sections is different. The difference between a 348‐mm scan length and three scans at a 160‐mm nominal beam width (480 mm) is 132 mm. The ratio of overlapping volumetric sections (132 mm) and scan length (348 mm) is 38%. However, the difference between a 352‐mm scan length and four scans at a 120‐mm nominal beam width (480 mm) is 128 mm. The ratio of overlapping volumetric sections (128 mm) and scan length (352 mm) is 36%. Therefore, it must be noted that the ratio of overlapping volumetric sections in the smaller nominal beam width is smaller than that in the larger nominal beam width. Conversely, the small intestine dose in the 320‐row MDCT unit does not correlate with the scan length and the number of rotations. The wide‐volume mode automatically adjusts the scan length by overlapping volumetric sections. Matsunaga et al.^14^ reported that operators must take into account the number of rotations, the beam width automatically determined by the scan length, and the placement of overlap between volumetric sections, because these parameters can greatly affect the radiation dose to the fetus and organs. Furthermore, the larger ratio of overlapping volumetric sections when using the larger nominal beam width should also be noted.

Scan lengths differ due to differences in fetal size. Therefore, it may not make sense to use the mathematical phantom in the ImPACT software because it does not scale for weight. Angel et al.^20^ reported that the abdominal circumference of a pregnant woman, but not the gestational age of the fetus, is significantly correlated with fetal dose. Facilities in Japan perform fetal CT in order to screen for skeletal dysplasia after an average of 30 weeks of gestation.^21^ In Japan, the mean abdominal circumference of a 28‐ to 32‐week pregnant woman has been reported to be 89–92 cm.^22^ Angel et al.^20^ also reported that the range of normalized fetal doses in pregnant patients with abdominal circumferences of 89–92 cm was 11.2–12.6 mGy/100 mAs. The results determined by the ImPACT software (12.0 mGy/100 mAs), calculated by Angel et al.,^20^ were equivalent to the normalized fetal dose in a pregnant patient with an abdominal circumference of 89–92 cm. Furthermore, Matsunaga et al.^16^ reported that the fetal dose differences between the thermoluminescent dosimeters and the ImPACT software measurements were <1 mGy in pregnant patients with an abdominal circumference of 90 cm. Therefore, the fetal dose in a pregnant patient with this abdominal circumference can be estimated using the mathematical phantom in the ImPACT software.

## 5 CONCLUSIONS

The approximate fetal dose may be estimated by multiplying the displayed DLP with a conversion factor (0.06 and 0.05 for 320‐ and 80‐row MDCT units respectively). As the scan length (176–348 mm) and the number of rotations (2–3) increased, the fetal doses and maternal effective doses increased, but the opposite was true for a further increase (scan length from 348 to 352 mm and the number of rotations from 3 to 4). It must be noted that the ratio of overlapping volumetric sections is larger when larger nominal beam widths are used.

## CONFLICTS OF INTEREST

The authors declare no conflict of interest.
